# Characterization of the complete mitochondrial genome and phylogenetic analysis of *Amanita franzii* (Amanitaceae, Agaricales)

**DOI:** 10.1080/23802359.2026.2694132

**Published:** 2026-07-13

**Authors:** Wen-Hao Zhang, Xiu-Juan Li, Yu-Xian Gao, Li-Ping Tang

**Affiliations:** ^a^ School of Pharmaceutical Sciences and Yunnan Key Laboratory of Pharmacology for Natural Products, Kunming Medical University, Kunming, China; ^b^ Yunnan College of Modern Biomedical Industry, Kunming, China

**Keywords:** Fungi, mitogenome, phylogeny, sect. *Phalloideae*

## Abstract

*Amanita franzii* is a non-lethal species of *Amanita* sect. *Phalloideae* belonging to the *A. pseudogemmata* subclade. In this study, we report its complete mitochondrial genome assembled from Illumina paired-end reads. The mitochondrial genome is 57,020 bp with a GC content of 22.27% and contains 15 conserved PCGs, three putative ORFs, two rRNA genes, and 27 tRNA genes. Phylogenetic analysis based on concatenated mitochondrial PCGs places *A. franzii* within sect. *Phalloideae*, where it forms a distinct lineage from the cyclopeptide toxin-producing clade. This study presents the first complete mitochondrial genome of *A. franzii* and provides a resource for evolutionary studies.

## Introduction

*Amanita* Pers. is one of the most taxonomically significant and intensively studied genera within Agaricales (Cui et al. 2018). Among its infrageneric groups, *Amanita* sect. *Phalloideae* has attracted particular attention because it includes many lethal species responsible for severe mushroom poisonings (Zhang et al. 2010; Chen et al. 2014; Tang et al. 2016). In recent years, the phylogenetic structure and taxonomic framework of this section have been progressively clarified through multilocus molecular phylogenetic studies (Cai et al. 2014; Cui et al. 2018).

Within this context, *Amanita franzii* Zhu L. Yang, Y.Y. Cui & Q. Cai 2018 was formally described in 2018 by Cui et al. based on a combination of morphological characters and multilocus phylogenetic evidence (Cui et al. 2018). The species is assigned to sect. *Phalloideae* and placed within the *Amanita pseudogemmata* Hongo 1974 subclade. Together with the lethal amanitas subclade and the *Amanita hesleri* Bas 1969 subclade, the *A. pseudogemmata* subclade constitutes three well-supported and phylogenetically independent lineages within sect. *Phalloideae* (Cui et al. 2018; Codjia et al. 2022). Notably, cyclopeptide toxins have not yet been reported from members of the *A. pseudogemmata* subclade, which forms a distinct lineage within sect. *Phalloideae*. (Cui et al. 2018, 2021; Codjia et al. 2022). However, mitochondrial genomic data for this subclade remain unavailable.

Mitochondrial genomes have proven to be powerful tools for elucidating fungal phylogeny and genome evolution (Li et al. 2020, 2023; Feng et al. 2024; Wang et al. 2025). Nevertheless, within *Amanita*, mitochondrial genomic characteristics across different subclades remain insufficiently explored, particularly in non-lethal species of sect. *Phalloideae*. In this study, we characterized the complete mitochondrial genome of *A. franzii* and assessed its phylogenetic placement within *Amanita* based on concatenated mitochondrial protein-coding genes (PCGs), providing new genomic resources for non-lethal species of sect. *Phalloideae*.

## Materials and methods

### Sample collection, DNA extraction and sequencing

**Figure 1.**
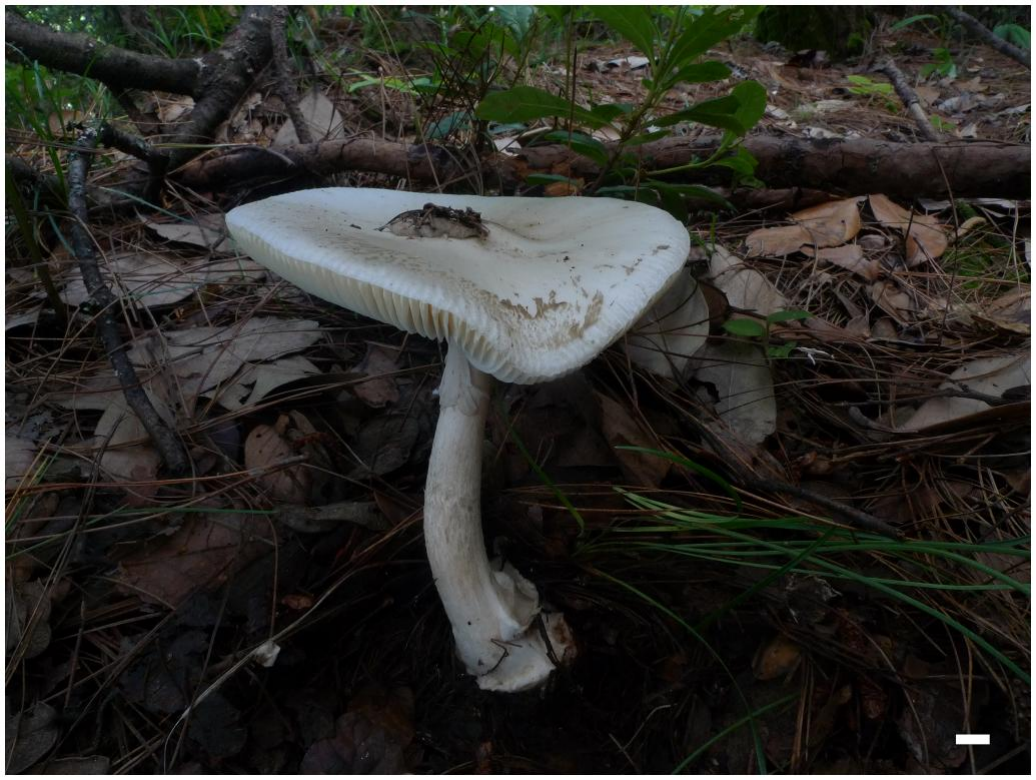
Basidiomata of *Amanita franzii* (MHKMU Tang-2364). Scale bar = 1 cm. Photograph by Liping Tang.

The specimen MHKMU Tang-2364 (Figure 1), identified as *A. franzii*, was collected on 23 August 2016 from Gaoqiao Town, Wuding County, Yunnan Province, China (25°39′17″N, 102°05′08″E; altitude 2550 m). The voucher specimen was deposited at the Mycological Herbarium of Kunming Medical University (MHKMU; contact person: Prof. Liping Tang, tangliping@kmmu.edu.cn) under the voucher number MHKMU Tang-2364.

Total genomic DNA was extracted from the desiccated specimen using the CTAB method (Doyle and Doyle 1987). A short-insert library with an insert size of 350 bp was prepared and sequenced by Novogene Co., Ltd. (Beijing, China) on the Illumina X Plus platform using the paired-end 150 bp sequencing strategy, generating approximately 5 Gb of raw data.

### Mitochondrial genome assembly and annotation

The mitochondrial genome of *A. franzii* was assembled from high-quality paired-end reads using GetOrganelle v. 1.7.7.1 with default parameters (Jin et al. 2020). Genome annotation was initially performed using the MITOS2 web server under genetic code 4 (Bernt et al. 2013). All predicted protein-coding genes were translated and manually inspected, and no anomalous internal stop codons were detected in any of the 18 PCGs. Protein-coding genes (PCGs) were further predicted with NCBI ORF Finder and validated by BLASTP searches against the NCBI non-redundant protein database (Bleasby and Wootton 1990). Ribosomal RNA genes were verified using RNAweasel (Beck and Lang 2010), and transfer RNA genes were identified with tRNAscan-SE v. 2.0 (Lowe and Chan 2016). Gene boundaries and start/stop codons were refined by alignment with homologous mitochondrial genes from published *Amanita* species using MAFFT in Geneious Prime 2024.0.5 (BioMatters Ltd., Auckland, New Zealand). The circular genome map was generated with OGDraw v. 1.3.1 (Greiner et al. 2019).

### Phylogenetic analysis

To clarify the phylogenetic position and taxonomic status of *A. franzii* within *Amanita*, phylogenetic analyses were conducted based on mitochondrial PCGs. The dataset comprised 15 *Amanita* species, with representatives of Lyophyllaceae Jülich 1982 and Tricholomataceae R. Heim ex Pouzar 1983 designated as outgroups. Fifteen mitochondrial PCGs, including atp6, atp8, atp9, cob, cox1–cox3, nad1–nad6, nad4L, and rps3, were extracted individually using Geneious Prime 2024.0.5. Each gene was aligned using MAFFT, and the resulting alignments were concatenated into a combined dataset for phylogenetic reconstruction. Phylogenetic trees were reconstructed using both Bayesian inference (BI) and maximum likelihood (ML) methods. BI was performed in MrBayes v3.2.7a (Ronquist et al. 2012) with four Markov chain Monte Carlo (MCMC) chains run for 1,000,000 generations. The first 25% of trees were discarded as burn-in. ML analyses were conducted in IQ-TREE v2 (Nguyen et al. 2015) under the edge-linked partition model, with branch support assessed using 1,000 ultrafast bootstrap replicates (Minh et al. 2013). Nodes were considered strongly supported when both ML bootstrap values (MLB ≥ 70%) and Bayesian posterior probabilities (BPP ≥ 0.95) were achieved.

## Results

**Figure 2.**
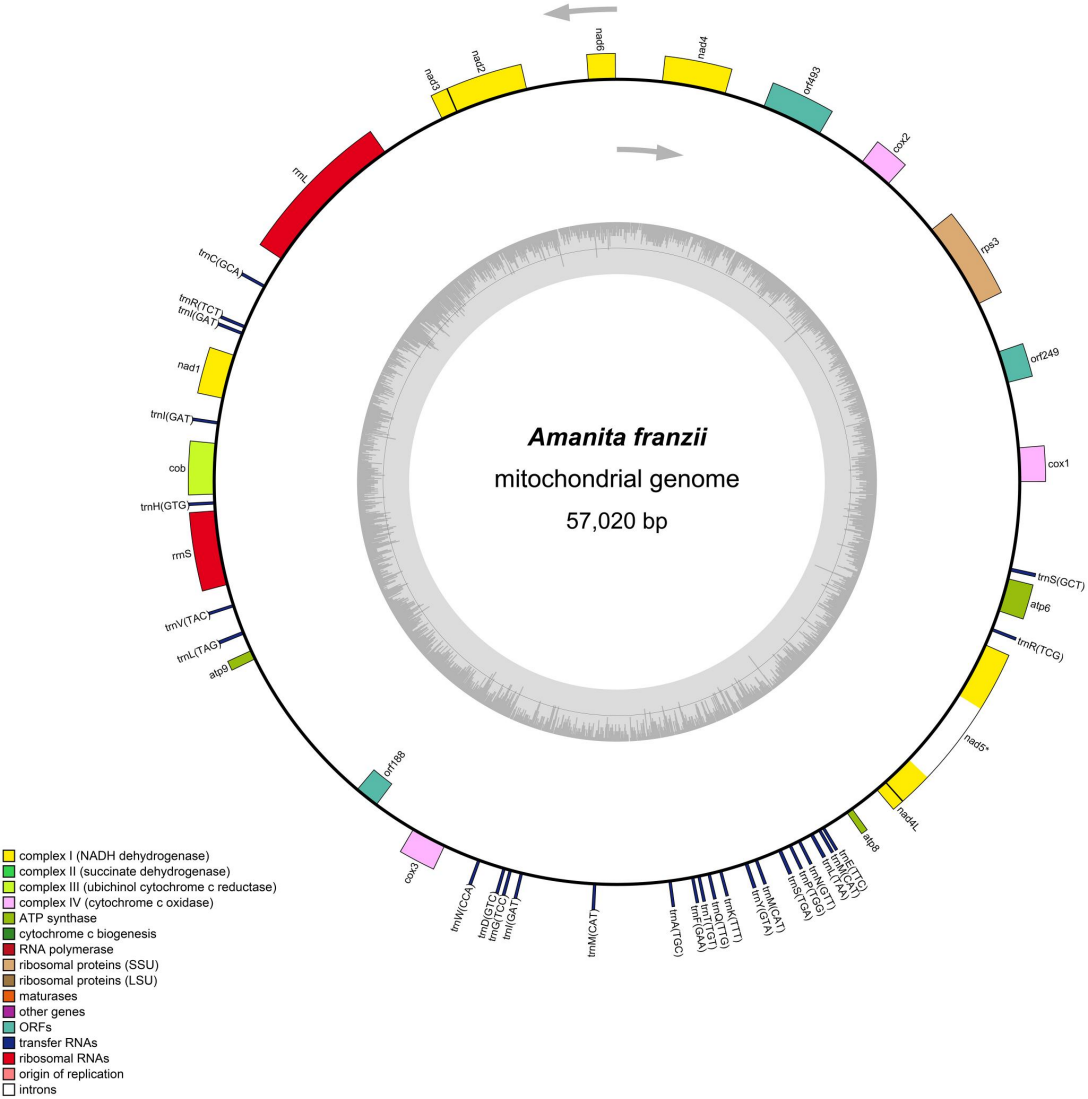
Circular map of the mitochondrial genome of *Amanita franzii*. Colored blocks denote different genes. Genes shown on the outer ring are located on the forward strand, while those on the inner ring are encoded on the reverse strand. The inner grey-scale bar plot indicates the GC content of the mitochondrial genome. Genes containing introns are marked with an asterisk (*).

Coverage depth analysis indicated that the mitochondrial genome of *A. franzii* was sequenced to an average depth of 11626.08 × (Figure S1). The complete mitochondrial genome of *A. franzii* was successfully assembled (Figure 2). The mitogenome is 57,020 bp in length and has a GC content of 22.27%. A total of 15 conserved mitochondrial PCGs were identified, including atp6, atp8, atp9, cox1, cox2, cox3, cob, nad1, nad2, nad3, nad4, nad4L, nad5, nad6, and rps3. Three additional putative ORFs were detected and showed significant similarity to homologous proteins in other *Amanita* species, although their functions remain unknown. The mitogenome also contains two ribosomal RNA genes (rns and rnl) and 27 transfer RNA genes (tRNAs).

**Figure 3.**
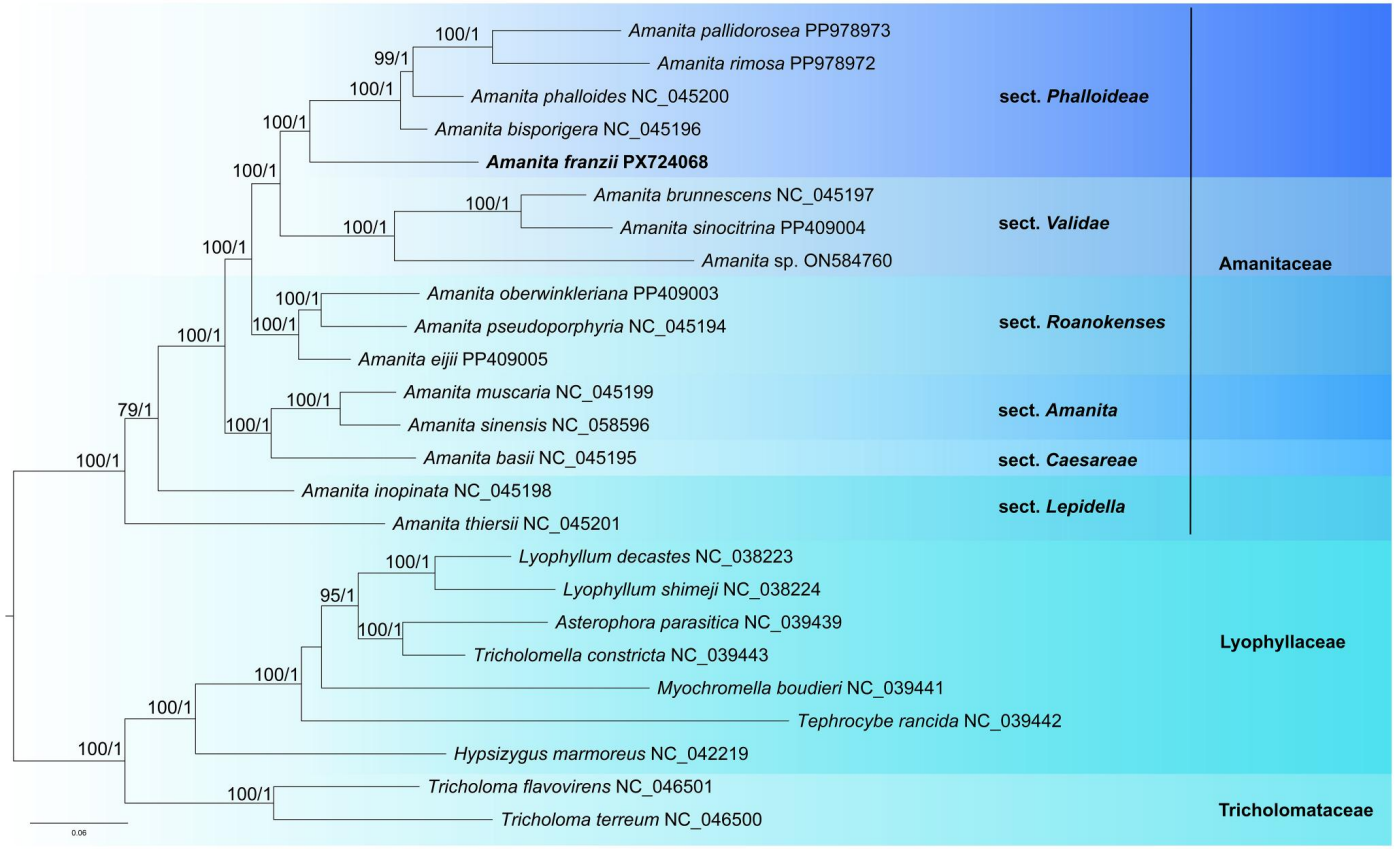
Phylogenetic relationships of 15 species inferred from concatenated mitochondrial PCGs under partitioned models. Node values indicate statistical support. Accession numbers of the mitochondrial genomes used in the analysis are provided after each species name. The following sequences were used: *Amanita pallidorosea* PP978973 (Wang et al. 2025), *Amanita rimosa* PP978972 (Wang et al. 2025), *Amanita phalloides* NC_045200 (Li et al. 2020), *Amanita bisporigera* NC_045196 (Li et al. 2020), *Amanita brunnescens* NC_045197 (Li et al. 2020), *Amanita sinocitrina* PP409004 (Wang et al. 2025), *Amanita* sp. ON584760 (unpublished), *Amanita oberwinkleriana* PP409003 (Wang et al. 2025), *Amanita pseudoporphyria* NC_045194 (Li et al. 2020), *Amanita eijii* PP409005 (Wang et al. 2025), *Amanita muscaria* NC_045199 (Li et al. 2020), *Amanita sinensis* NC_058596 (unpublished), *Amanita basi*i NC_045195 (Li et al. 2020), *Amanita inopinata* NC_045198 (Li et al. 2020), *Amanita thiersii* NC_045201 (Li et al. 2020), *Lyophyllum decastes* NC_038223 (Li et al. 2020), *Lyophyllum shimeji* NC_038224 (Li et al. 2020), *Asterophora parasitica* NC_039439 (unpublished), *Tricholomella constricta* NC_039443 (unpublished), *Myochromella boudieri* NC_039441 (unpublished), *Tephrocybe rancida* NC_039442 (unpublished), *Hypsizygus marmoreus* NC_042219 (Wang et al. 2019), *Tricholoma flavovirens* NC_046501 (Li et al. 2020), and *Tricholoma terreum* NC_046500 (Li et al. 2020).

Phylogenetic analyses based on the concatenated sequences of 15 mitochondrial PCGs showed that *A. franzii* is placed within sect. *Phalloideae* (Figure 3). Notably, *A. franzii* formed a distinct lineage separate from cyclopeptide toxin–producing species, including *Amanita bisporigera* G.F. Atk. 1906, *Amanita pallidorosea* P. Zhang & Zhu L. Yang 2010, *Amanita phalloides* (Vaill. ex Fr.) Link 1833 and *Amanita rimosa* P. Zhang & Zhu L. Yang 2010. The inferred phylogenetic relationships are congruent with those reported in previous studies, further demonstrating that mitochondrial genome data provide high resolution for elucidating evolutionary relationships within *Amanita*.

## Discussion and conclusions

This study reports the first complete mitochondrial genome of *A. franzii*. Coverage depth varied across the mitochondrial genome (628×–15,750×); however, coverage remained consistently high throughout the genome, supporting the reliability of the assembly. Phylogenetic analyses based on concatenated mitochondrial PCGs placed *A. franzii* within sect. *Phalloideae* and recovered it as a member of the *A. pseudogemmata* subclade, consistent with previous phylogenetic inferences based on nuclear markers (Cui et al. 2018; Codjia et al. 2022).

The availability of the *A. franzii* mitogenome provides the first mitochondrial genomic resource for the *A. pseudogemmata* subclade and expands current mitogenomic sampling within sect. *Phalloideae*. Incorporating mitochondrial genome data from such lineages will facilitate future studies on the evolutionary relationships within this section.

*Amanita* currently comprises three subgenera and eleven sections (Cui et al. 2018). To date, mitochondrial genomes have been reported for only six sections, viz. sect. *Amanita*, sect. *Caesareae*, sect. *Phalloideae*, sect. *Roanokenses*, sect. *Validae* and sect. *Lepidella*, while the other five sections remain unexplored at the mitogenomic level (Li et al. 2020; Wang et al. 2025). To gain deeper insights into the evolutionary history of *Amanita*, additional mitochondrial genomes from a broader range of species will be required in future studies.

## Supplementary Material

Supplemental Material

## Data Availability

Genome sequence data supporting the findings of this study are openly available in GenBank (NCBI) under accession number PX724068. The associated BioProject, BioSample, and Genome records are available in the National Microbiology Data Center (NMDC), China, under accession numbers NMDC10020250, NMDC20418749 and NMDC60221925, respectively.
