# Supplementary figures and images for: Characterization of the complete mitochondrial genome and phylogenetic analysis of Amanita franzii (Amanitaceae, Agaricales)
Source: Mitochondrial DNA B Resour. 2026 Jul 13;11(8):936–40. doi: 10.1080/23802359.2026.2694132 (PMC13366642; doi:10.1080/23802359.2026.2694132)

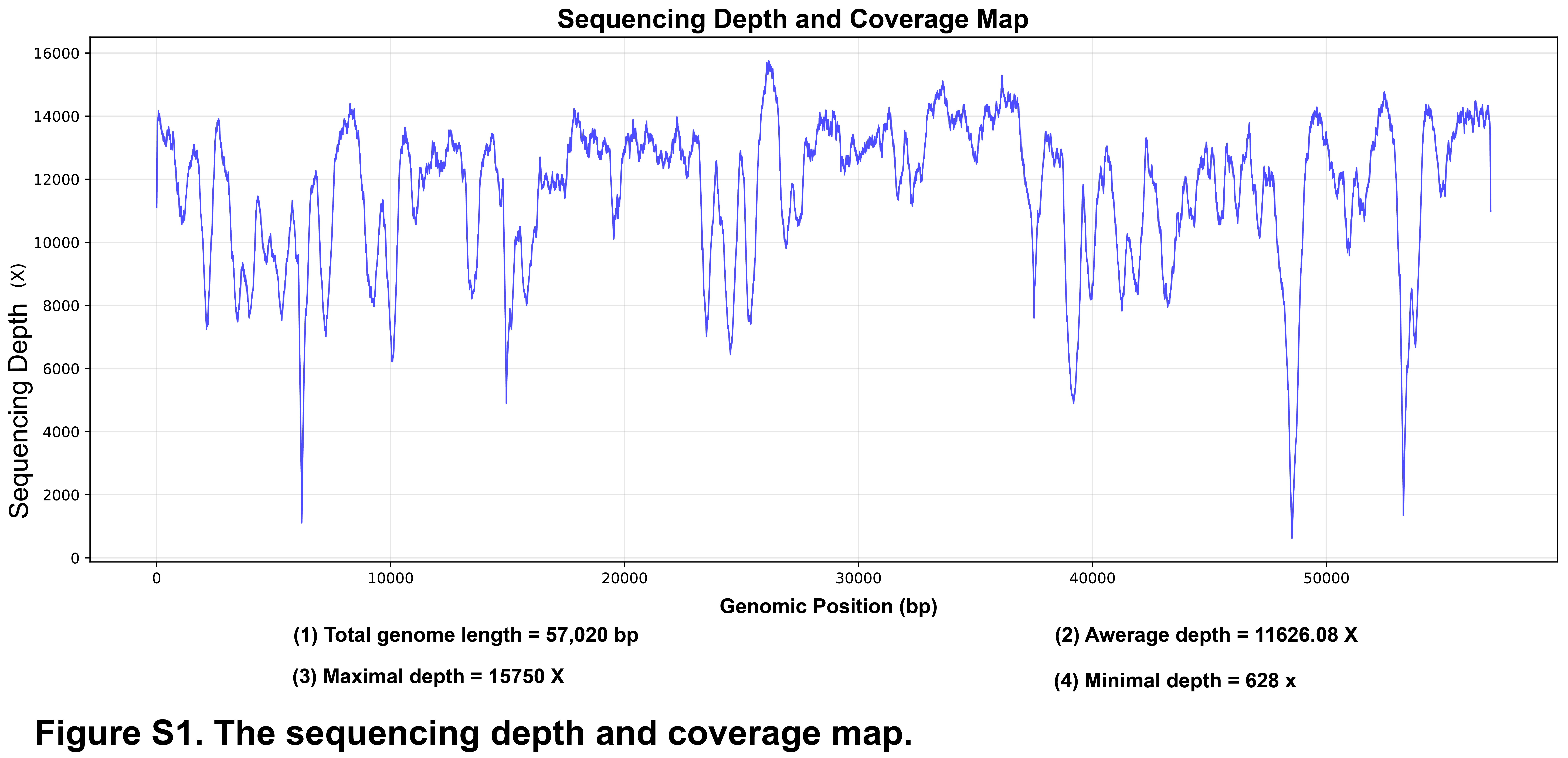

Supplement: Supplemental Material [file TMDN_A_2694132_SM6362.jpg]
